# Impact of sensory-based food education in kindergarten on willingness to eat vegetables and berries

**DOI:** 10.3402/fnr.v59.28795

**Published:** 2015-12-09

**Authors:** Ulla Hoppu, Mira Prinz, Pauliina Ojansivu, Oskar Laaksonen, Mari A. Sandell

**Affiliations:** 1 Functional Foods Forum, University of Turku, Turku, Finland; 2 Department of Biochemistry, Food Chemistry and Food Development, University of Turku, Turku, Finland

**Keywords:** food education, sensory, children, kindergarten, vegetables, berries

## Abstract

**Background:**

Children use all of their senses when exploring new foods, and sensory-based food education provides new possibilities for promoting healthy dietary habits.

**Objective:**

To evaluate the effect of sensory-based food education activities on children's willingness to eat test samples of selected vegetables and berries.

**Design:**

Two kindergartens in Hanko, Finland, participated in the study and the subjects were children aged 3–6 years, divided in the intervention (*n*=44) and control (*n=*24) kindergarten. In the intervention kindergarten, five sensory-based food education sessions focusing on vegetables and berries were implemented, once per week for 5 weeks. A tasting protocol was performed with the children at baseline and after the intervention. The willingness to eat (5 different vegetables and 3 Finnish berries) was categorised. Parents also filled in a questionnaire on the children's food preferences at home.

**Results:**

In the intervention kindergarten, the willingness to eat the samples increased significantly (*p*≤0.001, Wilcoxon and Friedman), while in the control kindergarten, no significant change was observed when all of the test samples were taken into account. The parental report of their children's preferences and children's actual eating of the test samples corresponded relatively weakly.

**Conclusions:**

Sensory-based food education activities may promote a willingness to eat vegetables and berries. Child-centred test methods are important for evaluating the effects of dietary interventions among children.

Children's low intake of vegetables and fruits is a nutritional challenge in many European countries (1, 2). Healthy dietary patterns in childhood, specifically diets rich in vegetables and fruits, have been demonstrated to be associated with a lower risk of cardiovascular diseases in adulthood (3). Kindergartens in Finland are served meals daily according to national dietary recommendations, and a variety of vegetables and fruits are served every day (4). However, the total intake of vegetables, fruits, and berries is low among Finnish children (5). Nordic wild berries are part of Finnish food culture, but their bitter, sour, and astringent taste may limit their consumption. Sensory properties, such as taste and flavour, have been shown to be critical factors in the preferences for vegetables and berries among children (6, 7). Food neophobia is also common among preschool-aged children and may be associated with lower consumption of vegetables and fruits (8).

New and innovative approaches exploiting sensory practices have been introduced in kindergarten and school settings to promote healthy eating (9). Sensory-based food education is a training concept based on sensory perception and experiences and their impact in learning processes related to food. Sensory education offers activities for the learning process via our senses by smelling, touching, hearing, watching, and tasting (10, 11). French ‘Glasses du Goût’ (Sapere taste education), developed for school-aged children, is a well-known sensory education method (12). In Finland, project funding has been available for training day care personnel and various sensory-based activities have been implemented in many kindergartens (13). The practical experiences have been encouraging, but there is a lack of documented scientific evidence on the effectiveness of these activities among kindergarten children.

The challenge in assessing food preferences and dietary intake among children is that most methods rely on parental reports that may be prone to parental subjective impressions and misreporting (14, 15). Parents rely on their opinion of food habits at home, and they may also have misunderstandings of how their children behave in meal situations at day care. Thus, objective child-centred methods to measure food acceptance, preferences, and consumption are important to develop and evaluate. Young children's cognitive and verbal skills need to be considered when developing new testing protocols (16). The choice, preparation, and presentation of test samples for children requires further consideration, and the testing location and research personnel may also have an impact on the test situation (17).

In the present study, sensory-based food education activities previously used in Finland were implemented in a new kindergarten with no previous experience with these activities. The main aim of the study was to evaluate the effectiveness of sensory-based food education in a kindergarten setting among children aged 3–6 years. To measure the programme impact, a child-orientated test protocol was developed to evaluate children's willingness to eat the test samples of vegetables and berries (primary objective of the present study). Furthermore, parent's opinions on their children's food preferences were compared to the children's willingness to eat the samples in the actual test situation (secondary objective).

## Methods

### Study population and protocol

This study was conducted in Hanko, located in southern Finland, with a total population of 9,100. Two kindergartens were chosen to participate in this study in collaboration with the Director of Early Childhood Education in Hanko. In one kindergarten, sensory-based food education activities were performed with the children (intervention), as described in detail in this article, while the other acted as a control with no activities. The same project researcher was responsible for the food education activities and the measurements with the children.

The study was targeted at children aged 3–6 years. In the intervention kindergarten, there were 44 children eligible for the study, and in the control kindergarten, 42 children were eligible. All of the parents of these children were sent a notice explaining the study and consent forms, and all of the parents of the 44 (intervention) and 24 (control) children provided written consent for their children to participate. Children could also refuse to participate or discontinue the activities and/or the test. The ethical committee of the University of Turku approved the study procedures.

### Questionnaire for the parents

At baseline, the parents completed a questionnaire that contained background data about the parents (year of birth, education, employment situation, and smoking status). Parents also completed a questionnaire including the child food neophobia scale (the Finnish version focusing on children) (18, 19). Parents were also asked to assess how much their child liked certain vegetables and berries (the same that were tested in practice) on a scale of seven different options (from fully agree to fully disagree) and complemented with the option ‘not served at home’. For data processing, responses that took on values 1–3 (1=fully disagreed, 2=rather disagreed, 3=slightly disagreed) were regrouped as disliked, while values 5–7 (5=slightly agreed, 6=rather agreed, 7=fully agreed) were grouped as liked. Value 4 ‘not known’ referred to responses that were not in agreement or disagreement.

### Measurements with the children

Measurements with the children were performed at baseline and after the intervention (5 weeks) in both kindergartens. The samples chosen for tasting contained a selection of vegetables (carrot, cabbage, swede, rucola, and romaine lettuce) and Finnish berries (bilberry, lingonberry, and sea buckthorn). The vegetable samples were cut in pieces (total sample weight 10 g in a plastic, transparent cup), and leafy vegetables were cut in strips (4–5 pieces in the cup). Frozen, whole bilberries and lingonberries (10 g sample) were placed in a cup and served frosty. The sea buckthorn juice sample was of 2 ml. The sea buckthorn was sampled as a juice due to its availability and because the juice is a common preparation of this berry in Finland. All of the samples were placed on a tray and the samples were stored in the refrigerator prior to the test. Good hygienic practices according to national food regulations were followed in the preparation and storage of the food samples. The children's food allergies were discussed and taken into account in all phases.

The measurements with the children were performed with the project researcher in a peaceful and isolated place in the kindergarten. All children attended the measurement individually, and the session lasted approximately 15–20 min. The instructions were explained to the children and the tray with the samples in plastic cups was placed in front of them. The vegetable samples were served first, followed by the berry samples. Children were allowed to freely explore the samples by seeing, smelling, and touching before tasting. They could pick the samples by hand, but a spoon was also offered. They were allowed to decide the order in which the samples were to be tasted. The results of the tasting were recorded immediately on the study form by the researcher. First, the researcher recorded if the child refused to taste the sample. If the child tasted the sample, the amount eaten was inspected and further categorised as the proportion of the sample eaten (categorisation: not tasted, tasted a bit, ate half of the sample, or ate the whole sample). This four-category variable is the main outcome measure of the present study and is defined by a single term, ‘willingness to eat’.

### Description of the sensory-based food education activities

In the intervention kindergarten, five different food education sessions were implemented, once per week for 5 weeks. Every child attended five food education sessions. There were usually three to four children (with a maximum of 7 children) in these groups. Activities were scheduled between 9.00 and 11.30 am, and the sessions lasted for 20–30 min. Activities were planned according to the sensory-based activities implemented previously in Finnish kindergartens (13). The contents of the sessions are described briefly in Table 1. They activated all five senses: taste, smell, sight, hearing, and the sense of touch. For example, smell was activated by odour bottles containing food-related odours. Pure taste samples (sweet, salty, sour, and bitter) were tasted in dilute water solutions. The sense of touch was activated by sensing different food samples in opaque fabric bags.

**Table 1:** Goals and tools of the sensory-based food education programme

Activating session	Goal of a session	Tools
1st ‘Familiarisation’	Explore and get familiar with own senses (sense of smell, touch, taste, hearing, and sight). Learning by playing evocative activating games	Sensory cards, puppet, cloth bags for touching, food samples (9), magnifying glass, odour bottles (7)
2nd ‘Applying to senses’	Observing food samples and surrounding with smelling, touching, hearing, looking, and tasting	Sensory cards, puppet, cloth bags with food samples (4), variety of food samples, magnifying glass, kitchen utensils, play shop, food cards
3rd ‘Sweet-sour’	Learning sweet and sour in food focusing on vegetables, fruit and berries with five senses	Sensory cards, food cards, puppets, activating fairy tale, variety of food samples, magnifying glass, kitchen utensils, play shop
4th ‘Bitter-salty’	Learning bitter and salty focusing on cooking with five senses	Sensory cards, variety of food samples, kitchen utensils, stories, salad materials
5th ‘Umami’	Learning umami and food talk focusing on food exploring with five senses	Sensory cards, variety of food samples, kitchen utensils, stories, variety of food samples, play shop, food posters

The sessions included the tasting and exploration of real food samples, concentrating on vegetables and berries. Of the samples tested in the test protocol, carrots, cabbage, swede, rucola, romaine lettuce, and lingonberries were included in the activities. In addition to tasting, food samples were investigated with a magnifying glass, they were smelled and touched, and the sounds of chewing were heard. Additionally, pictures of the foods were used and children could play with the food cards and identify different vegetables and berries. All activities, including tasting the foods, were voluntary, but children were generally highly curious, and most of them were willing to taste the foods in these sessions.

### Statistical analyses

Background characteristics of the groups were compared with chi-square test (categorical variables) and *t*-test (continuous variables). The non-parametric related-samples Wilcoxon signed rank test was used to analyse the median of differences for willingness to eat comparing the baseline measurement (m1) and the post-intervention measurement (m2) within the control and intervention groups independently. Non-parametric related-samples Friedman's two-way analysis of variance was used to analyse the willingness to eat (comparing m1 and m2) in the control and intervention groups independently. All food samples, both together and separately (*n*=8), were analysed to study the effect of the sensory-based activities. Statistical analyses were performed using IBM SPSS Statistics 21.0 (IBM Corporation, Armonk, NY, USA).

## Results

**Fig. 1:**
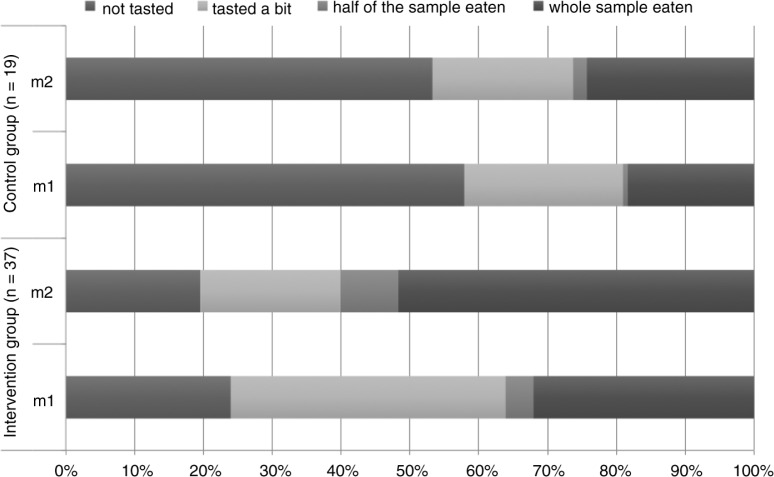
Willingness to eat all of the test samples together in the intervention and control groups at baseline (m1) and after the intervention (m2).

The characteristics of the intervention and control children are presented in Table 2. The differences in the background variables between the groups were not statistically significant. The results of the test protocol implemented in kindergartens at baseline and after the intervention are presented in Table 3. When the willingness to eat (total amount of all samples) was compared, there was a significant difference both in the distribution (*p*<0.001, Friedman's) and the median of differences (*p<*0.001, Wilcoxon) in intervention kindergarten between measurements at baseline and after the intervention. There was no significant change within the control group between measurements. Specifically, the proportion of samples completely eaten increased significantly (*p<*0.001) in the intervention group. The willingness to eat all test samples together is also illustrated in Fig. 1.

**Table 2:** Background characteristics of the intervention and control children and their mothers

	Intervention	Control	*p* *
N consents	44	24	
Girls (*n*, %)	22 (50%)	14 (58%)	0.40
Age of the child, years (mean, SD)	5.1 (0.8)	4.7 (0.9)	0.054
*N* responded to questionnaire	39	22	
Children's neophobia score, mean (SD)	34.0 (10.0)	39.8 (14.8)	0.069
Age of the mother, years (mean, SD)	34.1 (5.7)	33.0 (7.0)	0.52
Maternal education, university or polytechnic	23%	32%	0.46
Maternal employment, working	74%	77%	0.80
Maternal smoking	26%	14%	0.31

* Categorical variables compared with chi-square test and continuous variables with *t*-test.

**Table 3:** Willingness to eat (%) test samples in the intervention (INT, *n*=37) and control groups (CNTR, *n*=19) at baseline (m1) and after the intervention (m2)

	Phase	Not tasted	Tasted a bit	Half of the sample eaten	Whole sample eaten	Wilcoxon, *p*	Friedman's, *p*
Carrot							
INT	m1	10.8	18.9	21.6	48.6	0.001	<0.001
	m2	5.4	8.1	5.4	81.1		
CNTR	m1	52.6	5.3	0.0	42.1	0.25	0.48
	m2	31.6	15.8	5.3	47.4		
Swede							
INT	m1	18.9	32.4	5.4	43.2	0.022	0.004
	m2	10.8	10.8	13.5	64.9		
CNTR	m1	52.6	31.6	0.0	15.8	1.0	1.0
	m2	63.2	10.5	10.5	15.8		
Cabbage							
INT	m1	21.6	37.8	0.0	40.5	0.039	0.074
	m2	21.6	13.5	5.4	59.5		
CNTR	m1	57.9	21.1	0.0	21.1	0.39	0.41
	m2	68.4	15.8	0.0	15.8		
Romaine lettuce							
INT	m1	27.0	35.1	0.0	37.8	0.17	0.061
	m2	21.6	21.6	5.4	51.4		
CNTR	m1	78.9	5.3	0.0	15.8	0.033	0.034
	m2	47.4	26.3	0.0	26.3		
Rucola							
INT	m1	27.0	54.1	2.7	16.2	0.048	0.108
	m2	29.7	21.6	16.2	32.4		
CNTR	m1	68.4	21.1	0.0	10.5	0.59	0.41
	m2	78.9	10.5	0.0	10.5		
Bilberry							
INT	m1	18.9	27.0	0.0	54.1	0.001	0.001
	m2	5.4	8.1	5.4	81.1		
CNTR	m1	52.6	10.5	0.0	36.8	0.49	0.70
	m2	42.1	21.1	0.0	36.8		
Lingonberry							
INT	m1	27.0	54.1	2.7	16.2	0.012	0.074
	m2	24.3	27.0	13.5	35.1		
CNTR	m1	52.6	42.1	0.0	5.3	0.17	0.41
	m2	52.6	26.3	0.0	21.1		
Sea buckthorn							
INT	m1	40.5	59.5	0.0	0.0	0.22	0.47
	m2	37.8	51.4	2.7	8.1		
CNTR	m1	47.4	47.4	5.3	0.0	0.11	0.41
	m2	42.1	36.8	0.0	21.1		
All samples							
INT	m1	24.0	39.9	4.1	32.1	<0.001	0.001
*n*=8×37 = 296	m2	19.6	20.3	8.4	51.7		
CNTR	m1	57.9	23.0	0.7	18.4	0.25	0.11
*n*=8×19 = 152	m2	53.3	20.4	2.0	24.3		

Statistical analyses (Wilcoxon and Friedman) were made within the group (INT and CNTR) by comparing the results of m1 and m2.

When the samples were inspected separately (Table 3), it was found that the willingness to eat carrots (*p*<0.001), swede (*p*=0.004), and bilberries (*p<*0.001) increased statistically (Friedman's) in the intervention group. There was also a significant difference in the median intake of carrots, cabbage, swede, rucola, bilberries, and lingonberries (Wilcoxon). In the control group (Table 3), only the willingness to eat romaine lettuce increased between measurements (*p=*0.033; Wilcoxon, *p=*0.034; Friedman).

**Fig. 2:**
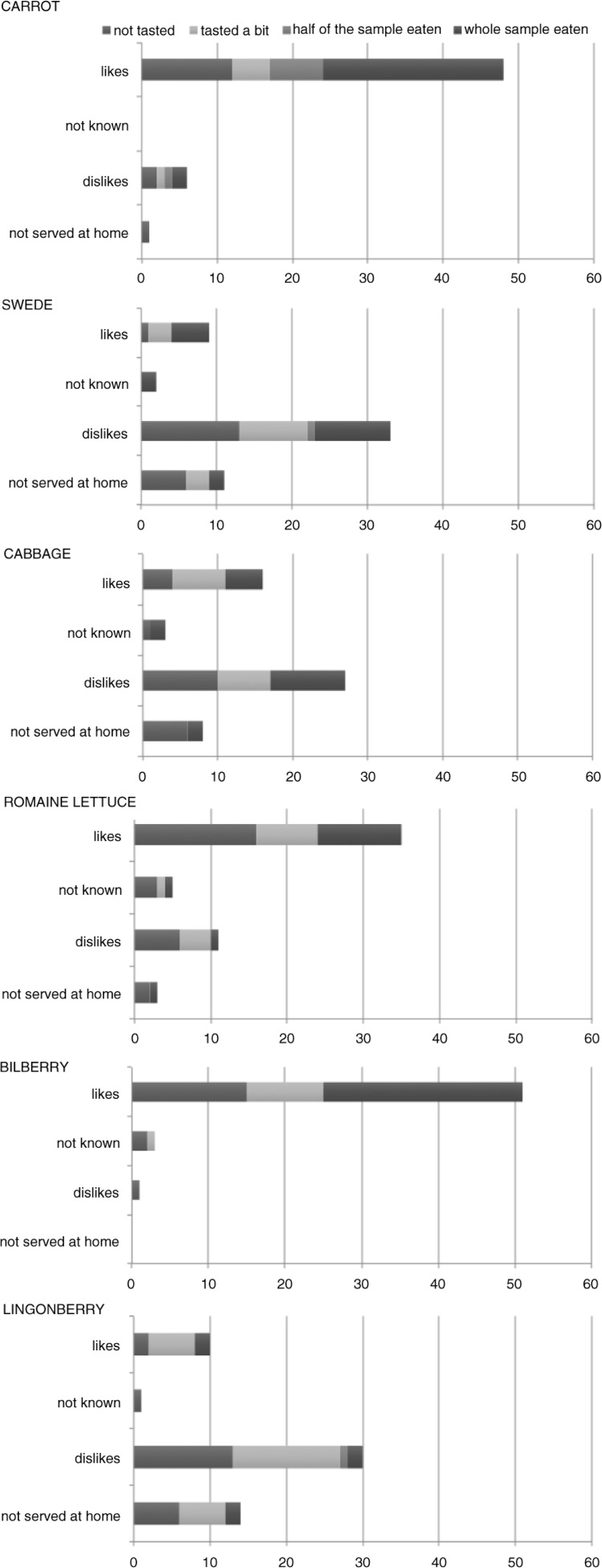
The preferences of children for specific vegetables and berries as reported by their parents and their intake in the test situation (baseline measurement, both groups together, *n*=55 families).

Figure 2, which depicts comparison of parent opinion and child behaviour, illustrates how parents reported their children's preferences for certain vegetables and how the children tasted or ate the samples in the test situation. The figure highlights that in many cases, the parental answers and children's behaviour did not interrelate with each other. For example, in the case of swede, cabbage, or lingonberries, many parents reported that their child disliked these foods; however, many children were willing to taste or even eat the sample in the test situation.

## Discussion

In the present study, sensory-based food education activities concentrating on vegetables and berries were employed in a kindergarten. These activities were shown to increase the willingness to eat test samples of vegetables and berries. The arrangement of sensory tests for small children is often laborious and time demanding, but the developed test procedure was found to be practical and easy to use in kindergarten. It was challenging to motivate the control group subjects in particular to participate in the study, and although the number of children was quite low, the results are promising. The period with activities was quite short (5 short sessions over 5 weeks) and the measurements were carried out shortly after the activity sessions were finished (within the next week). Thus, an evaluation of the long-term impact requires further studies with larger study populations and with longer intervention and follow-up periods.

There are very few scientific reports on the effectiveness of sensory-based activities, including all five senses, among children. Previous studies have concentrated in school-aged children and have found positive effects in promoting the willingness to taste novel foods among children aged 8–10 years (11), and in reducing neophobia among children aged 8–11 years (19). Sensory education also improved the children's skills in describing the sensory properties of foods (20). Among Dutch schoolchildren aged 9–12 years, the taste lessons were shown to result in an increased number of foods known and tasted in the intervention group, as reported by the children themselves in the questionnaire after 4 weeks of the intervention (21). Dazeley and Houston-Price (10) reported the results of a non-taste sensory activity programme among children aged 12–36 months in the UK. They found that in a meal-time taste test, children touched and tasted more of the vegetables that they had been familiarised with in activities. Additionally, some other programmes promoting healthy eating for preschool-aged children, such as *Color Me Healthy*, have included some sensory components, but the effects of sensory activities in multicomponent interventions are difficult to assess (22).

It is well-known that repeated exposure to vegetables may increase intake (23, 24), and frequent exposure during sensory activities might thus promote the tasting and eating of new vegetables. Additionally, visual exposure alone, such as looking at picture books about fruits and vegetables, may influence the willingness to taste these foods (25). Recently, Wagner et al. (26) reported that toddlers’ food preferences were correlated with their liking of the odour of the food. Sensory characteristics, including texture, have been shown to be important to children, and therefore different preparation methods of vegetables may be related to children's preferences (27). Thus, more information on the combination of all senses in children's food perceptions and further sensory-based food education activities are required.

Kindergarten offers a good operational environment for sensory-based education. The pedagogical skills of the personnel help to develop activities appropriate for different age groups. The activities implemented in this study are easy and cost little to incorporate into daily pedagogical activities. Peer modelling in kindergarten may promote children's acceptance of new foods (28). Food services at day care may also be involved in food education, and daily lunch and snack times offer constant possibilities for sensory experiences. The day care practices and meal services at day care differ between countries, and thus new food education activities need to be developed for local conditions. Involving children in meal preparation (29) and gardening (30) may offer possibilities for motivational activities to influence children's attitudes, knowledge, and behaviours related to vegetables and fruit. Thus, sensory-based food education could be part of the normal daily activities in kindergartens and could promote healthy eating in the long run.

We also observed that there were clear discrepancies between the parental report on their child's liking and the child's actual eating of the test samples. Parents rely on their opinion of the children's eating habits and meal situations at home, and although the test situation differs from this, the differences are still notable. This result is consistent with findings in previous studies, for example, Liem et al. (31) found that only 39% of mothers were able to correctly predict their children's most preferred flavour in a preference ranking task of five ice-cream flavours. They also found that older children (5–10 years) reported more stable flavour preferences than younger children (3–5 years) (31). Vereecken et al. (32) compared children's and parents’ responses on young children's fruit and vegetables preferences and they found that the agreement between parents and their children was rather low to moderate. Thus, relying only on parental reports may distort the reality of children's preferences and consumption of foods.

Previous studies aiming to increase and measure children's preferences and consumption of vegetables have used various tasting protocols. The choice of test samples and measuring methods depends on the aims of the study (33). The arrangement of the test situation also depends on local practices in kindergartens and the place (in separate room or in the lunch room), time of day (in the morning or during snack time), and personnel (familiar kindergarten personnel or study personnel), as well as whether the measurements are performed individually or in group need to be considered (17). Thus, careful planning and pilot testing of methods suitable for the study question, age group, food culture, and other factors are required of future studies. Clear indicators and outcomes should be defined for evaluating interventions (34). The method developed and tested here seemed to be motivational for children, practical, precise enough and easy to use and thus suitable for testing Finnish children in a day care setting. The strengths of the present study are also the presence of a control group and the measurements with the same test protocol both at baseline and after the intervention. Because children's preferences and behaviours will change as they grow older, it is important to have a control group when evaluating the effectiveness of sensory-based education, especially for long-term interventions.

The limitation of the present study is the low number of participants, but for practical reasons we were able to include only two kindergartens in this small-scale study. As the kindergartens are located in different areas, the background variables of families may also differ at baseline. This a common challenge in all real-life interventions carried out in kindergarten settings. The two kindergartens participating in the study represented typical day care settings in small Finnish towns, but the generalisability of the results on a larger scale needs to be further studied.

Interventions in kindergarten may increase children's willingness to eat vegetables served in day care. However, the total consumption of vegetables cannot be markedly improved if the supply is not increased at home. Parents are responsible for the foods offered at home and the availability of vegetables and fruits at home (35), as well as serving as parental role models (36), and have been demonstrated to be important determinants of children's food intake. Therefore, parental involvement and motivation to increase vegetable consumption should have a role in further interventions. Additionally, Finnish adults consume fewer vegetables and fruits than recommended and many adults may not enjoy the taste of vegetables, such as their bitterness, thereby limiting their intake (37). Food neophobia may also reduce vegetable intake of adults (38). Therefore, various sensory-based activities could also be developed for the whole family to encourage both adults and children to diversify and increase their vegetable intake.

In conclusion, food education activities in kindergarten may promote children's willingness to eat vegetables and berries. Further research on effective methods to implement sensory-based food education and scientific research examining the impact of these activities on children's dietary choices are warranted.
